# What are the perceived learning needs of Australian general practice registrars for quality prescribing?

**DOI:** 10.1186/1472-6920-10-92

**Published:** 2010-12-09

**Authors:** Rola Ajjawi, Jill E Thistlethwaite, Parisa Aslani, Nick B Cooling

**Affiliations:** 1 Faculty of Medicine, Nursing and Health Sciences, Monash University, Notting Hill, VIC 3168, Australia; 2 Institute of Clinical Education, The University of Warwick, Coventry, UK CV4 7AL, UK; 3 Faculty of Pharmacy, The University of Sydney, NSW 2006 Australia; 4 GP Training Tasmania, New Town, Tasmania 7008, Australia

## Abstract

**Background:**

Little is known about the perceived learning needs of Australian general practice (GP) registrars in relation to the quality use of medicines (QUM) or the difficulties experienced when learning to prescribe. This study aimed to address this gap.

**Methods:**

GP registrars' perceived learning needs were investigated through an online national survey, interviews and focus groups. Medical educators' perceptions were canvassed in semi-structured interviews in order to gain a broader perspective of the registrars' needs. Qualitative data analysis was informed by a systematic framework method involving a number of stages. Survey data were analysed descriptively.

**Results:**

The two most commonly attended QUM educational activities took place in the workplace and through regional training providers. Outside of these structured educational activities, registrars learned to prescribe mainly through social and situated means. Difficulties encountered by GP registrars included the transition from hospital prescribing to prescribing in the GP context, judging how well they were prescribing and identifying appropriate and efficient sources of information at the point of care.

**Conclusions:**

GP registrars learn to prescribe primarily and opportunistically in the workplace. Despite many resources being expended on the provision of guidelines, decision-support systems and training, GP registrars expressed difficulties related to QUM. Ways of easing the transition into GP and of managing the information 'overload' related to medicines (and prescribing) in an evidence-guided, efficient and timely manner are needed. GP registrars should be provided with explicit feedback about the process and outcomes of prescribing decisions, including the use of audits, in order to improve their ability to judge their own prescribing.

## Background

The practice of quality use of medicines (QUM) aims to reduce prescribing errors and possible adverse effects in patients. QUM encompasses: selecting management options wisely; choosing suitable medicines if a medicine is considered necessary; and using medicines safely and effectively [1]. Specific indicators of quality prescribing have been categorised into three basic types: structural, process and outcome [2].

Prescribing errors in clinical practice are common, accounting for around 2-14% (median rate 7%) of prescriptions or medication orders in most studies. This prevalence is similar in UK [3], US [4], and Australian studies [5], and across hospital [3] and primary care settings [6,7]. Errors are more likely to occur in junior doctors [8] but are still prevalent in senior general practitioners and hospital consultants [3]. Errors in prescribing can be attributed to a variety of factors including individual, environmental and organisational such as lack of knowledge, insufficient training, workload and communication [3,9-11]. They are also difficult to quantify due to prevalent problems with available data, error definition and study design [12].

While prescribing errors are common, they may not result in actual adverse outcomes for the patient. Conversely adverse outcomes might not be due to poor prescribing [5]. Most prescribing errors are minor, and are detected by pharmacists or nurses, especially in the hospital setting, preventing patient harm [3,13,14]. However a small proportion of adverse outcomes are severe and safety nets are less available in some settings such as general practice (GP).

In the few studies examining the quality of prescribing of Australian general practitioners [6,15], there appears to be a need to improve prescribing quality. A variety of educational approaches, including the use of quality indicators, has been suggested [2]. However, it is difficult to change the prescribing habits of experienced doctors [9]. Thus there is the expectation that educating junior doctors to prescribe in a QUM fashion is a more effective intervention. This study was designed in part to address this gap by focusing on perceived needs of GP registrars and their supervisors. The research questions were:

1. What existing QUM programs (including resources and educational activities) do GP registrars use?

2. What prescribing difficulties do GP registrars experience during training?

## Methods

We analysed Australian GP registrars' training needs in QUM in the second half of 2008, funded by the National Prescribing Service (NPS). Multiple stakeholders were involved (GP registrars and supervisors, medical educators) and multiple methods of data collection (survey, focus group and interview) were used [16]. Ethical approval was obtained from the University of Sydney ethics committee.

We developed an online questionnaire to explore the uptake of QUM programs related to prescribing by GP registrars based on questions identified in the literature, our experience with the target population and our research questions. The survey was edited to maximise ease of use, piloted with five GPs and the wording of questions further refined. The survey used open and closed questions and was organised in four parts: 1) use of information resources and learning activities, 2) barriers and enablers to QUM, 3) knowledge of the NPS, and 4) general demographics information. Registrars were recruited through an invitation email sent by directors of training at regional training providers (RTPs) across Australia to all registrars in their training scheme. Reminder emails were sent and incentives (book vouchers) offered to improve response rates.

Additional data collection activities, semi-structured interviews and focus groups, occurred at three RTPs - GP Synergy (formerly SIGPET; Sydney), General Practice Training Tasmania (GPTT) and Gippsland Education and Training for General Practice (GetGP; Victoria). These RTPs were chosen to widen diversity of the sample by including urban and rural GP registrars and supervisors across three states. Focus groups were scheduled to occur following organised educational sessions at each RTP. Invitation emails and information statements were emailed to registrars in advance with the researcher's contact details if they wished to participate. The researcher (RA) who undertook recruitment and collected the data was independent from the RTPs. Interview guides were prepared which included questions about resources used when prescribing, educational activities attended and perceived difficulty in prescribing.

Survey data were exported from Survey Monkey^© ^to an excel spreadsheet. Quantitative data were analysed descriptively. Audio recording of interviews and focus groups were transcribed, checked for accuracy and imported into qualitative data management software. Data analysis was informed by a systematic framework method [17] involving a number of stages starting with iterative reading of transcripts by two researchers (RA and JT), who identified recurrent concepts (codes) raised by respondents. They negotiated a thematic framework outlining the content of participants' data. This framework was used to code the entire data set. Codes were grouped together into a smaller number of sub-themes and similar codes collapsed.

## Results

### Participant demographics

Sixteen RTPs distributed the survey; with 1154 GP registrars contacted. The number of respondents was 225 (response rate 19.5%). Respondent demographics (Table 1) were similar to the GP registrar population at the time the survey was conducted. Three to six registrars participated in each of seven focus groups at GP Synergy, GPTT and GetGP (n = 34 registrars). Nine GP supervisors and medical educators participated in semi-structured interviews.

**Table 1:** Demographics of survey respondents compared with national data held by Australian General Practice and Training (AGPT) for Semester 2, 2008

		Respondents	Population
**Gender**	Female (%)	68	63

**Graduates**	Australian Universities	80	75

**Age**	Average	35	35

	Range 25-36 years old (%)	75	64

**Location**	Urban (RRMA1-2)^1^	41	46

	Rural & Remote (RRMA3-7)	59	49

**Training^2^**	% Basic registrar training	35	12

	% Advanced registrar training	15	25

	% Subsequent registrar training	35	30

^1 ^Rural, remote and metropolitan area classification can be found at: http://www.aihw.gov.au/ruralhealth/remotenessclassifications/rrma.cfm

^2 ^Basic training registrars (6 months full time equivalent (FTE) training in GP), Advanced training registrars (12 months FTE), Subsequent training registrars (18 months FTE)

### Uptake of QUM programs

The most commonly used resources for prescribing were MIMS (including MIMS accessed through software prescribing systems), Therapeutic Guidelines (TG) and Australian Medicines Handbook (AMH). This finding was confirmed by survey and focus group data. These three resources were also the most valued and highly rated as 'very useful'.

Formal education activities related specifically to prescribing were limited (Table 2). RTPs provided specific prescribing information during orientation activities. Prescribing was also indirectly discussed in some workshops about diseases or case studies. GP Networks conducted evening topic-based workshops regularly. These often ran in conjunction with a NPS representative, and at times pharmacists, and contained information about medicines and pharmaceutical management.

**Table 2:** GP registrar participation in educational activities related to prescribing in the last three months

Activity	Number who participated^1^
**Tutorials held at the workplace**	106 (47%)

**Regional Training Provider workshop**	99 (44%)

**Case study**	60 (36%)

**Academic detailing**	70 (31%)

**GP Network/Division-based education activities**	64 (28%)

**Pharmaceutical workshop**	55 (24%)

**National Prescribing Service workshop**	42 (18%)

**Clinical audit**	14 (6%)

**Other: exam or personal study/reading**	11 (5%)

^1 ^These percentages add to more than 100% as respondents were able to indicate more than one response.

### Difficulty in prescribing

GP registrars reported several difficulties related to prescribing, grouped into four categories (Table 3).

**Table 3:** GP registrars' reported difficulties in relation to prescribing

1) Prescribing inherently involves complex decision-making processes
2) Finding appropriate information, efficiently at the point of care

3) Judging how well one is prescribing

4) GP context-specific prescribing: the transition from hospital to GP prescribing

#### 1) Prescribing is a complex decision making process

Registrars perceived prescribing as complex and influenced by a variety of people including peers, supervisors, other GPs, pharmacists and pharmaceutical representatives. Not surprisingly, *GP supervisors *played a key role in influencing and promoting GP registrars' prescribing either through active strategies such as discussion and coaching with patient cases as they arose or, less frequently, in organised case-discussion sessions. GP supervisors also acted as role models; registrars reporting they would learn from their supervisors' prescribing 'habits'.

You just pick on your supervisor's prescribing habits and you copy them. (SIGPET fg1)

*Pharmacists *were a commonly reported source for highlighting prescribing mistakes, and were sought out for specific information including cost of medicines, and dosage. *Peers and colleagues *were also a source of information, through informal networking and discussion in the practice or at continuing education events.

We have a big group of doctors in our practice - like seventeen doctors and often we discuss different medications and just compare our own experiences [with] different types of medications. (GPTT fg2)

The frequency with which *pharmaceutical representatives *were seen depended on how often they were invited to visit the practice by GPs. This varied from daily to weekly or monthly lunch visits.

The drug reps that sort of visits, well they visit us very often, maybe two or three times a week with coffees [laughter] and at the same time educate us about all the latest drugs and the latest research. (SIGPET fg2)

The influence of the representatives can be seen in the choice of medicine prescribed in response to, for example, medicine samples.

[It's] really helpful them giving out samples and you can start with them so if the patient is responding well then I can continue. (SIGPET fg1)

However, GP registrars were cautious about the objectivity of this information and its marketing focus. They expressed concern about being adversely influenced by the pharmaceutical representatives, adopting different strategies for dealing with them, such as avoidance or asking targeted questions.

Prescribing complexity was particularly evident in variations in prescribing and conflicting opinions about medicines. Registrars commented on the difficulty in negotiating or dealing with these conflicting opinions with their supervisors, particularly the difficulty working with GPs who were perceived as 'out of date' in prescribing.

Bring the GPs of the 70's and 80's up to date and in tune with current knowledge. Help them to understand the value of well informed doctors who base prescribing on evidence or at least rational consensus guidelines. (R72 survey)

The difficulties associated with GP registrars reconciling conflicting opinions with their GP supervisors when there is a power imbalance was acknowledged by the medical educators:

Being a registrar is a really complicated thing. Your boss is your teacher, that's actually a conflict of interest. (SIGPET ME3)

#### 2) Finding appropriate information related to prescribing when needed

GP registrars reported difficulties in staying up to date with medicines information, including knowing what resources are available and their value.

That's hard for me if I was going to start a patient on some drug it would be hard for me to come up with actual practical information about what sort of side effects that they could have. (SIGPET fg1)

Finding appropriate information about the costs of medicines to patients was commonly identified as difficult. Providing patients with the wrong information about cost of medicines can reduce patient adherence and possibly confidence.

I find it very difficult to get through the relationship between costs to the patient, find out information in a timely fashion and just understanding regulations and the PBS [Pharmaceutical Benefits Scheme]. (SIGPET fg1)

Managing the sheer volume of information about medicines and interpreting complex resources were also discussed.

There's so much [information] online and stuff, like it's hard to know which one's the most useful or which one's the recommended, so you end up wasting your time trying everything. (SIGPET fg2)

Newer registrars reported feeling frustrated and anxious about looking up resources in the presence of the patient.

Patients thinking I don't know anything if I need to look something up. (R3 survey)

#### 3) Judging one's prescribing

The opening question in registrar focus groups was: 'How do you know if you are prescribing well?' Registrars at times seemed at a loss to answer. Their responses indicated that some factors they looked for were improvement or resolution of the patient's symptoms with minimal side effects.

The patient gets better and we reach ... a therapeutic goal like for example with LDL [low density lipoprotein] and they're not getting side effects and they're happy with the medication then I think that that's a medication which I think I've prescribed well. (SIGPET fg1)

Contact from the local pharmacist was often reported as a way of identifying errors in prescribing.

We don't really know if we're not prescribing well unless our pharmacist calls us. But otherwise, I mean, I don't know whether I'm doing it right or not. (SIGPET fg2)

#### 4) GP context-specific prescribing

The transition from hospital to prescribing in the GP context was a difficult aspect for new GP registrars. They reported concerns about feeling isolated, having difficulty understanding the system, having fewer resources and back up compared with hospital and having less time within which to make prescribing decisions. The frustration expressed with understanding the system is apparent in the following quotes:

I hate that feeling of being isolated in general practice and just having a few computer or written resources to tap into. (GPTT fg2)

I need a simple handout or workshop in person (not online) explaining what PBS is, cost of medicine to patients, how to use resources, how to write a script and repeats - most of us come from hospital and have no idea how to do this! (R60 survey)

The diminished general practice 'safety net' was discussed by both GP registrars and medical educators. This referred to the reduced opportunities for checking of scripts by pharmacists, nurses and consultants compared with hospitals. The added responsibility was acutely obvious to the registrars.

In the hospital we are always getting picked up by pharmacists, you know, daily pretty much, you get very helpful comments made about drug interactions that you haven't noticed. (GPTT fg1)

Registrars and medical educators also mentioned time management and the pressure of making a decision on the spot:

Some registrars feel pushed into making a decision about giving a drug there and then [during consultation] ... and often therefore will guess or have a bit of a go at it, rather than ... wait for another appointment. (GPTT fg ME)

## Discussion

GP registrars reported learning to prescribe through social and situated means. Learning opportunities were mostly in the workplace (learning on the job, looking up resources), and opportunistic (asking the supervisor, informal discussion with peers). Pharmacists were identified as helpful in identifying prescribing errors, this is in keeping with data on interception of prescribing errors by pharmacists in hospital [3] and primary care settings [14,18]. There were few organised educational activities related to prescribing specifically beyond orientation sessions and self-directed online case-studies.

Four main areas of perceived difficulty were expressed by GP registrars. The prescribing process is not simply about choosing a medicine and writing a prescription. With the aging population, increased use of polypharmacy and increasing complexity of pharmacology in modern drugs, prescribing is becoming increasingly difficult and carries a greater risk of drug interactions and adverse reactions [19]. GPs do limit the scope of options considered in prescribing [20]. In the case of GP registrars, options presented to patients may be restricted by registrars' knowledge, which affects adherence. Conversely, if the GP registrar is aware of the options they are more likely to adopt a shared decision making approach with patients rather than a paternalistic model.

Another aspect of complexity we identified related to the multiple sources of influence and variations in prescribing. Influences on prescribing were similar to those reported in the literature about GP prescribing [21,22]. The significant influence of the supervisor was evident and is in keeping with USA residents' use of sources of information in ambulatory care. They tended to rely upon consultant physicians for information [23]. Residents only engaged in a more formal search for information when presented with a clinical dilemma beyond the scope of their attending physician's clinical experience. This highlights the essential role of the GP supervisor in learning to prescribe.

The notion of conflict and complexity in the GP registrar-supervisor relationship was evident and has been explored previously from the supervisors' [24] but not the registrars' perspective. How prescribing decisions are negotiated between registrars and their supervisors, in particular when there is a conflict in opinion, is an area for further research.

Registrars identified pharmaceutical representatives as sources of information and influence on their prescribing. GPs have been found to rely on medication guides published by pharmaceutical companies [21,25]. British GPs in 2003 identified the pharmaceutical industry as the most important influence on the use of a new medication [21]. There have been changes to representative access to GPs in the UK but not yet in Australia. This reliance is problematic as research from the Netherlands suggests increased contact with representatives translates to poor quality prescribing by solo GPs [26]. In the meantime, educational tools to assist GP registrars to critically appraise representatives' information are desirable.

The registrars found it difficult to stay up to date with medicines information, including knowing what resources are available and their value. Managing the sheer volume of information about medicines and interpreting complex resources were challenging. Such difficulties are likely to extend beyond GP registrar training, as doctors receive a plethora of information about new medicines, changes in evidence-based guidelines and to the Pharmaceutical Benefits Scheme. Physicians have been shown to be more likely to seek information if this was reliable, easily and quickly accessible [27]. Ensuring access to objective and well presented information at the point of care, plus assisting GP registrars to critically identify valuable resources is important to achieve QUM.

Difficulty was identified in judging the quality of prescribing. Self-assessment does not appear to be a stable, global skill that is easily acquired or developed but rather it is situationally bound and context specific [28]. Feedback from reliable others (here GP supervisors, medical educators and pharmacists) has been suggested as necessary to inform the ability to judge actions and decisions [28-30]. This refers to feedback not just on the outcomes of decisions, which occur ad hoc when pharmacists call or patients return to see the GP, but also on the decision making process itself [31]. Academic detailing and prescribing audits with specific feedback have been found to improve prescribing and would assist GP registrars as a source of external and objective feedback [32-34]. What is surprising is that despite this evidence to support their use, prescribing audits are not mandatory during GP training in Australia.

In this study local pharmacists were a valued source of feedback to GP registrars on their prescribing errors but were under-utilised in training programs and educational sessions. We recommend improving interprofessional collaboration. A randomised controlled trial showed that increasing input from clinical pharmacists reduced the use of inappropriate medicines in an elderly population [35] in particular through the integration of pharmacists into GP settings [36]. Finally, structured case-based discussions with supervisors could also be used to address this gap.

Difficulties in the transition from hospital prescribing to prescribing in the GP setting highlight the context specific nature of decision making [37]. The influence of contextual factors on prescribing decisions has been documented [20,38]. GP registrars perceived the loss of a 'safety net' compared with prescribing in hospital, where consultants would dictate prescribing and nursing and pharmacy staff would check scripts prior to administering medicines. Indeed one Australian study found that interns take sole responsibility for only one-fifth of the prescriptions they chart which may impact significantly on their opportunity to acquire the skills necessary to become independent, rational prescribers [39]. In addition, hospital patients are more readily monitored for adverse effects in comparison to community-based patients.

Structured educational activities related to prescribing and the GP context, are indicated beyond orientation sessions. Stronger vertical integration between medical school programs and vocational training may be one way of softening the transition gap. To this end the NPS in partnership with representatives from medical schools created structured online case studies available free to all medical students across Australia [40]. It is based on the *World Health Organization Guide to Good Prescribing *and a similar program is being developed for junior doctors. Specific coaching on how to manage decision-making in the GP context may also be indicated. This assertion is further supported by evidence that prescribing errors are caused by deficiencies in complex, contextual knowledge rather than the underpinning theory or declarative knowledge commonly taught in undergraduate programs [3].

This study adds to existing data demonstrating a widespread problem with prescribing for which there appears to be no consensus on the instructional designs that will ensure patient safety through efficient and effective prescribing [9]. Lack of feedback is a really important aspect that is uncovered by this work which is probably true of a lot of prescribing throughout the healthcare sector. We have provided recommendations for the specific difficulties expressed by the registrars who participated in this study based on existing evidence (albeit patchy) and educational principles.

### Limitations

There has been little research specifically targeting GP registrars' learning needs in relation to prescribing. The strength of mixed methods [41] and multiple sources of data collection [42] were utilised for this needs analysis. This study is the only one known to examine this population at a national rather than regional or local level, and to seek input from a range of stakeholders. Despite considerable effort in recruiting participants for the survey including reminders and incentives the response rate was relatively low (20%), though on-par with response rates from research with the Australian GP population [43]. Consistency of findings with the different sources of data collection is reassuring although caution is required in generalising as the response rate was low.

In this research we sought GP registrars' experienced difficulties with prescribing considering the breadth of QUM resources and education opportunities currently available to them. While educational interventions based solely on felt needs are less reliable than normative or expressed needs - when well conducted they can lead to significant behaviour change in doctors [44]. Asking key experts such as medical educators about the needs of junior doctors can increase the normative component of a needs assessment. Future research could seek to measure prescribing competence in the GP setting as part of a comprehensive needs analysis.

## Conclusion

GP registrars used familiar, trusted and readily accessible resources at the point of care for their prescribing. Uptake of QUM educational activities was dependent on provision in the workplace and by RTPs. Despite many resources being expended on the provision of guidelines, decision-support systems and training, GP registrars expressed difficulties related to QUM. Prescribing was addressed during orientation yet GP registrars reported difficulty in the transition to GP prescribing. Helping GP registrars learn how to judge their prescribing decisions and outcomes as well as managing the flow of information about medicines is indicated by our findings.

## Competing interests

The authors declare that they have no competing interests.

## Authors' contributions

All authors contributed to the design of the research, the first author collected and analysed the data. The second author aided in the data analysis. All authors contributed to the writing of the manuscript and have read and approved the final manuscript.

## Funding

This project was funded by the Australian Government Department of Health and Ageing through National Prescribing Service Limited.

## Pre-publication history

The pre-publication history for this paper can be accessed here:

http://www.biomedcentral.com/1472-6920/10/92/prepub
